# Clinical Lectures on Diseases of Nurses and Infants

**Published:** 1846-06

**Authors:** 


*Clinical Lectures on Diseases of Nurses and Infants.* By PROFESSOR TROUSSEAU.

*Sclerema.*—The first case to which we will direct your attention, is that of an infant, aged twenty-five days, and affected with induration of the cellular tissue. The mother is dying of what may be called a sort of puerperal farcy; when she came to the hospital, abscesses were forming in the limbs, and a collection of pus had accumulated in the left knee, with considerable pain and febrile excitement. She was improved so much during the few days which followed her admission, by the exhibition of one-twelfth of a grain of calomel every hour, that we thought her saved. Salivation was established at the expiration of the second day, all the morbid symptoms had yielded, and fluctuation could no longer be felt in the diseased joint. Since then a poultice, with leaves of datura stramonium, opium, and camphor, has been applied to the knee, but a sudden relapse has taken place, and her present state leaves us but little hope of saving her life. For several days the infant took the empty breast; the want of proper food, and perhaps his unsuccessful efforts to draw from his mother a sustenance which she could not yield, disordered the gastric functions, caused diarrhœa and vomiting, which milk and magnesia have to a certain extent allayed; but yet the weakness of the child continued, he grew gradually colder, and on touching his body this morning, (April 6th,) we find the legs and thighs in a marked state of induration. The child's cry is still vigorous, but a fine crepitus may be heard in the lungs. This will be a fatal case; true sclerema is not cured, and death is generally the result of the extension to the external viscera of that rigidity which you notice in the external organs. In many cases of this malady, the incapacity of the lung to perform its functions is obvious, for the infant's head hangs over his chest, and a bloody saliva runs from his mouth. Most cases of sclerema, I might say all, are fatal; a difference of opinion on this point can only arise from errors of diagnosis, which may be accounted for in the following manner:—It is common to find children three or four months old, after diarrhœa or erysipelas, affected with limited œdema and hardness, which may be dispelled; but when, during the first month of life, without any functional lesion of the skin or any internal phlegmasia to explain the induration, a child becomes hard, he will surely die. M. Charcellay compares sclerema to the anasarca which sometimes follows scarlatina. He states that in both the urine is albuminous, and that both have their origin in a morbid condition of the kidneys. It is difficult to obtain the urine of so young an infant, but the research is an interesting one, and we will endeavour to ascertain its truth.

*Tubercular Disease in Children.*—Dr. Louis has stated that in childhood tubercles became frequent only between the ages of two and eight, and diminish in frequency from that period to puberty. This assertion could only have been made at a time when no special hospital existed for the treatment of diseases of new-born infants. During seven years, says Professor Trousseau, I have been attached to the care of infants at Necker's Hospital, and I distinctly assert that, under two years, more infants die of tubercular consumption than of all the other causes of dissolution put together. It is a circumstance which must not be forgotten, that infants may be affected with tubercular disease, and yet present none of those external appearances which we generally refer to the diathesis. As an illustration of this statement, we may bring forward the case of the infant who died of meningitis yesterday:—He was aged seven months, and was a remarkably fat, healthy, and lively-looking child. Both the parents were in the enjoyment of robust health; the child was suddenly seized with meningitis, and the malady remarkable besides by the manifestation of the characteristic red stain of the skin; the alternations of improvement which it presented, and the *removable* paralysis by which it was accompanied, was fatal in the space of five days. And I now present to you the lungs, spleen, liver, and intestines, studded with myriads of tubercles. On the surface of the cerebral envelopes, also, you may perceive numerous granulations strewed like a fine sand. It is not impossible that meningitis was in this case determined by the presence of these granulations; but I call upon you to remark that these granulations all occupy the convex surface of the cerebrum, and the anatomical changes resulting from the meningitis, are all, on the contrary, on the inferior aspect of the brain—a fact to be explained by the remark that whatever may have been the determining cause of meningitis, it always presents the same symptoms, progress, and anatomical changes. The chief conclusion I wish to draw from this case is, to caution you, when you meet with meningitis in very young children, although they may present none of the appearances of consumption, not to hasten to assert that the cerebral disorders have not originated in the deposition of tubercular matter. In a recent memoir, M. Rilliet states that meningitis often presents in children premonitory signs of very long duration, occurring three and even four months before the outbreak of the malady. These prodromic symptoms would be crossness, somnolency, feverishness, and various functional signs of cerebral suffering. In our opinion, M. Rilliet's observations have borne too exclusively on cases of *tubercular* meningitis. Now, it is well known that most of these are at the same time accompanied by visceral tuberculisation, and I am inclined to think that the crossness, erratic fever, &c., looked upon by M. Rilliet, as prognosticating meningitis, are only the symptomatic expression of a tubercular diathesis. Besides, in some cases of inflammation of the brain induced by the presence of tubercular granulations, we find sometimes only three or four granulations, forming so very small an organic lesion, that we cannot reasonably grant them the power of producing premonitory symptoms of any kind.

*Rheumatic Paralysis of Nurses.*—The disease I am about to describe is one which had never attracted my notice before 1841, and which I have never met with but in nurses. Do not attach any importance to the name I have given to it; I have used the designation only for want of a better. You may observe it in a woman at present lying in the wards. She was confined eight months ago, and continued in perfect health, nursing her child, for the next six months, when she was suddenly affected with the disease which has brought her to the hospital. Very often during the day, the patient is seized in one of her limbs with a sensation of numbness and formication, followed by spasmodic contraction and loss of power of the extremity; the symptoms last a few minutes, and reappear soon after in another limb, or in the tongue, throat, or eyes; in the latter case vision becomes indistinct, a ligature applied round a limb at once produces the spasmodic contractions. These are more frequent and severe in bed, and in the sitting posture. The patient's general health is good, but the frequent repetition of the symptoms has weakened her. The first time I met with this disease I was very much embarrassed. The patient had formerly suffered from rheumatism, and I fancied that her present symptoms might perhaps be attributed to the same unknown rheumatic element, so prone to migration. I bled her, and she was cured. Since that time I have met with the same symptoms in eight or nine nurses, and in none had rheumatism preceded them, so that I still remain very much in the dark as to the intimate nature of the disease. However, venesection proves in all cases sufficient to achieve a cure. The nurse at present in the wards had not passed, for the last two months, a single day without numerous attacks. We bled her yesterday morning, and none have occurred since. The blood is very fibrinous. At any rate, should you meet with similar cases, gentlemen, do not become alarmed about the state of the spinal chord; be convinced that the most serious (apparently) functional disorder of the nervous system may exist without any anatomical alteration of the nervous centres. I need only advert to nervous asthma, which is so frightful to behold, and yields so readily to the inhalation of stramonium, and to fits of hysteria, to impress strongly this point on your memory.—*Lon. Med. Times.*

